# Fetal androgen exposure is a determinant of adult male metabolic health

**DOI:** 10.1038/s41598-019-56790-4

**Published:** 2019-12-27

**Authors:** Katarzyna J. Siemienowicz, Panagiotis Filis, Sophie Shaw, Alex Douglas, Jennifer Thomas, Sally Mulroy, Forbes Howie, Paul A. Fowler, W. Colin Duncan, Mick T. Rae

**Affiliations:** 1 School of Applied Science, Edinburgh Napier University, Edinburgh, EH11 4BN UK; 2 Institute of Medical Sciences, School of Medicine, Medical Sciences & Nutrition, University of Aberdeen, Aberdeen, AB25 2ZD UK; 3 MRC Centre for Reproductive Health, The University of Edinburgh, Edinburgh, EH16 4TJ UK

**Keywords:** Developmental biology, Disease model, Physiology, Metabolism, Metabolic diseases, Dyslipidaemias, Endocrinology, Endocrine system and metabolic diseases

## Abstract

Androgen signalling is a critical driver of male development. Fetal steroid signalling can be dysregulated by a range of environmental insults and clinical conditions. We hypothesised that poor adult male health was partially attributable to aberrant androgen exposure during development. Testosterone was directly administered to developing male ovine fetuses to model excess prenatal androgenic overexposure associated with conditions such as polycystic ovary syndrome (PCOS). Such *in utero* androgen excess recreated the dyslipidaemia and hormonal profile observed in sons of PCOS patients. 1,084 of 15,134 and 408 of 2,766 quantifiable genes and proteins respectively, were altered in the liver during adolescence, attributable to fetal androgen excess. Furthermore, prenatal androgen excess predisposed to adolescent development of an intrahepatic cholestasis-like condition with attendant hypercholesterolaemia and an emergent pro-fibrotic, pro-oxidative stress gene and protein expression profile evident in both liver and circulation. We conclude that prenatal androgen excess is a previously unrecognised determinant of lifelong male metabolic health.

## Introduction

Prenatal programming of health and disease is driven by maternal/fetal endocrine alterations^1,2^, maternal nutritional status^3^ and maternal/fetal chemical insults^4^. During development, interactions between endogenous hormones, underlying endocrine disease and/or environmental exposure to toxicants, such as endocrine disrupting compounds (EDC)^5^ impact upon postnatal health, and thus the incidence of chronic disease^6^, with attendant economic burdens^7,8^.

The linkage between adult health and altered prenatal androgen exposure is robust. Androgens, secreted by developing testes^9^ and other organs^10^ promote masculinisation in target tissues expressing androgen receptors. In addition to pathologies of insufficient androgen exposure, for example hypospadias^11^, excess exposure may also occur, for example, in offspring of hyperandrogenaemic women with polycystic ovary syndrome (PCOS) or congenital adrenal hyperplasia^12^. Human data and animal experimentation has demonstrated aetiological contributions of prenatal androgen overexposure to the development of PCOS in female offspring^13^, linking the reproductive phenotype with metabolic phenotypes, including insulin resistance (IR), obesity and dyslipidaemia. This is underscored by morphometric markers of increased prenatal androgens in women with PCOS and their daughters^14,15^, and male range umbilical cord blood androgen concentrations in daughters of women with PCOS^16^. Indeed animal models designed to recreate PCOS-associated pathologies utilise over-exposure of female fetuses/pregnant dams to androgenic steroids^1,17–25^.

Male offspring from human PCOS pregnancies also develop hyperinsulinaemia and dyslipidaemia, predicting potentially increased risk of cardiovascular disease^26–28^. Elevated cholesterol is an early marker of metabolic dysfunction in pubertal sons of PCOS patients^29^. Early metabolic changes in PCOS sons are likely to be independent of gonadotropin and sex steroid levels although they have elevated anti-Müllerian hormone (AMH) concentrations during childhood^30^.

We hypothesised that androgen excess during male fetal development increases the risk of developing liver dysfunction and dyslipidaemia. The aims of the study were, in an animal model of prenatal androgen excess: **1**. Determine whether dyslipidaemia observed in PCOS-patient male relatives may have early-life-environment contributions. **2**. Comprehend hepatic mechanisms behind prenatally programmed dyslipidaemia. **3**. Determine circulating proteins reflective of prenatally programmed liver dysfunction prior to overt disease symptom development.

We discovered that fetal androgen excess was linked to adolescent male hepatic dysfunction/dyslipidaemia, elucidated mechanisms underpinning fetal androgen-excess programming of postnatal hepatic dysfunction, and identified circulating markers reflective of such programming in adolescence.

## Results

### Prenatal androgen treatment replicates human male PCOS-offspring characteristics

**Figure 1:**
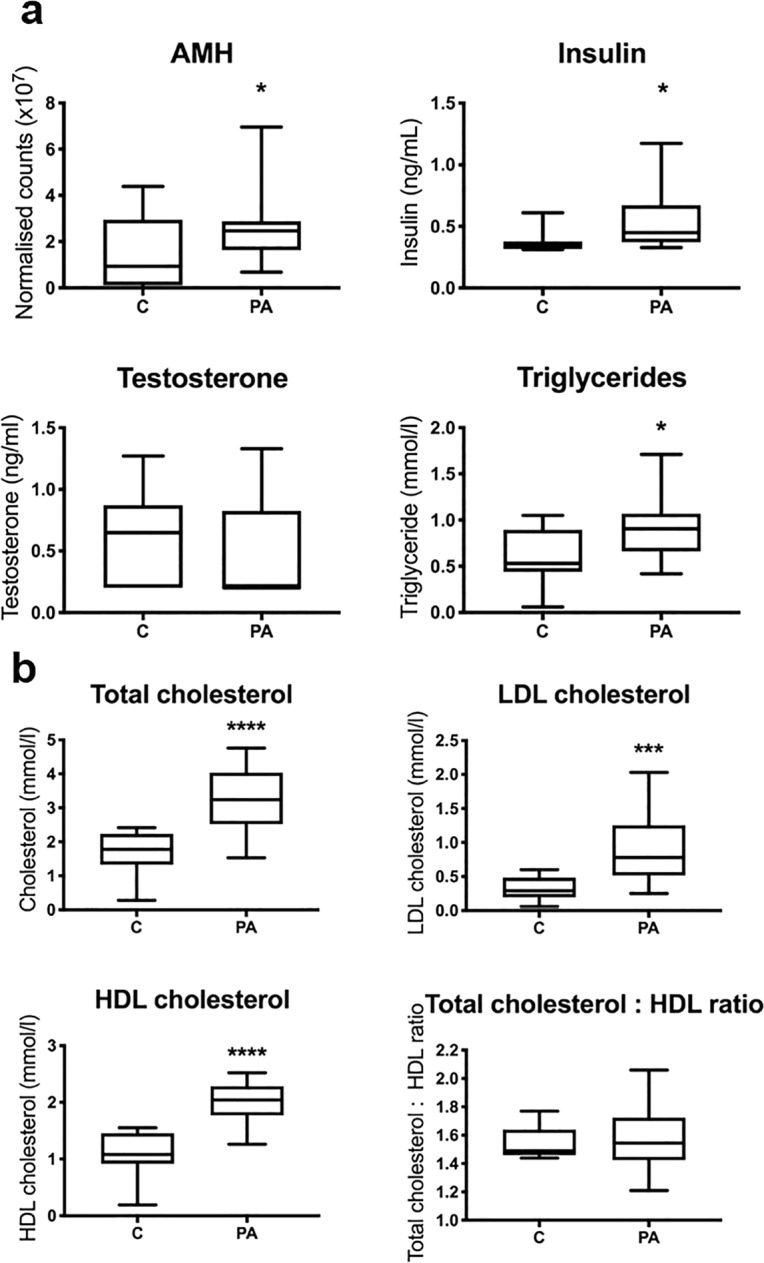
Prenatally androgen exposed males exhibit characteristics typical of pre- and post-pubertal sons of PCOS women. (**a**) Plasma characterisation: Samples from control (C; n = 14) and prenatal androgen excess (PA; n = 14) were collected at 6 months postnatal age from male offspring and assessed for common characteristics of pre- and pubertal sons of PCOS women. Prenatally androgen treated male offspring had significantly elevated circulating AMH, insulin and triglycerides, with no alteration in terms of testosterone concentrations. **(b)** Plasma cholesterol profiles: Prenatally androgen treated male offspring had significantly elevated total, LDL and HDL cholesterol concentrations as compared to vehicle control animals. Differences were analysed by unpaired, two-tailed t-test. (*P < 0.05; **P < 0.01; ***P < 0.001; ****P < 0.0001). Box plot whiskers are lowest and highest observed values, box is the upper and lower quartile, with median represented by line in box.

Circulating biomarkers characteristic of the sons of women with PCOS were examined in male sheep from control and prenatally androgen treated pregnancies. AMH and insulin were significantly elevated in adolescent prenatally androgen treated males (*P* < 0.05), but testosterone concentrations were comparable with control offspring (Fig. 1a). Plasma from prenatally androgen treated sheep therefore replicated circulating biomarkers of PCOS patients first degree male relatives^26–30^.

### Evidence of dyslipidaemia

Adolescent prenatally androgen treated males had increased plasma triglycerides (0.90 ± 0.08 mmol/l vs 0.58 ± 0.07 mmol/l; *P* < 0.05) (Fig. 1a) and elevated total cholesterol concentrations (3.27 ± 0.25 nmol/l vs 1.7 ± 0.16 mmol/l; *P* < 0.0001) (ovine reference range 1.3–1.96 mmol/l^31)^ compared with control offspring indicative of hypercholesterolaemia (Fig. 1b). Both LDL cholesterol (*P* < 0.001) and HDL cholesterol (*P* < 0.0001) concentrations were increased in adolescent prenatally androgen treated males when compared to controls (Fig. 1b).

### Prenatal programming of hepatic lipid metabolism

The hepatic transcriptome analysis revealed 15,134 expressed genes for analysis after low count transcript removal, with 1,084 differentially expressed genes in adolescent life between control and prenatally androgen treated livers (FDR adjusted *P* < 0.05). Hepatic proteome analysis quantified and identified 2766 proteins, with 408 differentially expressed in adolescent life between control and prenatally androgen treated livers (*P* < 0.05). Of the 449 plasma proteins quantified and identified, 96 were differentially expressed in adolescent prenatally androgen treated males compared to control animals (*P* < 0.05). Collectively, bioinformatics analysis indicated the prenatally-programmed dysregulation of pathways involved in lipid metabolism, metabolism of cholesterol, sterol synthesis, cholesterol synthesis and lipid homeostasis, together with prediction of altered cholesterol concentrations (Table 1), manifesting in plasma as hypercholesterolaemia (Fig. 1b).

**Table 1:** Bioinformatic characterisation of prenatally androgen exposed males.

Pathway	P-Value	Direction	Molecules
***Lipid metabolism***			
Metabolism of cholesterol	9.1 × 10^−14^	**↓**	31 genes
Sterol synthesis	1.08 × 10^−13^	**↓**	28 genes
Cholesterol synthesis	2.5 × 10^−11^	**↓**	23 genes
Lipid homeostasis	6.24 × 10^−7^	NP	29 genes
Disorder of lipid metabolism	4.46 × 10^−5^	**↑↑**	17 proteins
Homeostasis of lipid	1.23 × 10^−5^	NP	14 proteins
Metabolism of cholesterol	2.37 × 10^−7^	NP	12 proteins
Synthesis of cholesterol	1.8 × 10^−4^	NP	7 proteins
Cholesterol concentration	6.7 × 10^−4^	**↑**	16 proteins
Lipid homeostasis	7.85 × 10^−5^	NP	7 p proteins
Reverse cholesterol transport	7.64 × 10^−5^	NP	3 p proteins
Efflux of cholesterol	4.62 × 10^−5^	**↑**	6 p proteins
Hypercholesterolemia	7.34 × 10^−4^	NP	4 p proteins
***Dysregulation of enterohepatic circulation and cholestasis***			
Altered bile acid synthesis	3 × 10^−3^	NP	4 proteins
Intrahepatic cholestasis	2 × 10^−2^	NP	8 genes
Cholestasis	1.5 × 10^−3^	NP	7 proteins
***Reduced hepatic detoxification potential***			
Oxidative stress	1.23 × 10^−6^	**↑**	7 proteins
Hydrogen peroxide metabolism	5.93 × 10^−3^	**↓**	8 proteins
Synthesis of reactive oxygen species	1.4 × 10^−3^	**↑**	24 proteins
Concentration of glutathione	3.3 × 10^−3^	**↓**	7 proteins
Biosynthesis of hydrogen peroxide	6.37 × 10^−4^	**↑**	5 p proteins
Metabolism of reactive oxygen species	1.62 × 10^−5^	**↑**	14 p proteins
Synthesis of reactive oxygen species	2.18 × 10^−4^	**↑**	12 p proteins
***Liver damage***			
Liver damage	6 × 10^−4^	**↑**	32 genes
Drug-induced liver disease	1.23 × 10^−6^	NP	9 genes
Hepatocyte proliferation	1.83 × 10^−4^	**↑**	20 genes
Increased cell death	2.37 × 10^−7^	**↑**	119 proteins
Fibrosis	5.9 × 10^−4^	**↑**	11 p proteins
Liver lesion	3.26 × 10^−6^	**↑**	54 p proteins
Hepatic injury	4.08 × 10^−4^	NP	7 p proteins
Liver cirrhosis	5.28 × 10^−4^	NP	7 p proteins
***Systemic effects***			
Morbidity and mortality	1.49 × 10^−9^	**↑**	236 genes
Diabetes mellitus	1.88 × 10^−7^	NP	23 p proteins
Diabetic complications	6.33 × 10^−5^	NP	8 p proteins
Occlusion of artery	1.45 × 10^−7^	**↑↑**	16 p proteins
Atherosclerosis	2.01 × 10^−7^	**↑↑**	15 p proteins

Dysregulated hepatic metabolic pathways and disease states associated with differentially expressed genes and proteins in adolescent prenatally androgenized males (p-protein = plasma protein) were highlighted. ↑/↓ = mild directionality prediction (Z score ≤ 2/−2); ↑↑/↓↓ = strong directionality prediction (Z score > 2/−2); NP = no prediction made.

### Prenatally programmed dysregulation of hepatic cholesterol synthesis, uptake and secretion pathways

**Figure 2:**
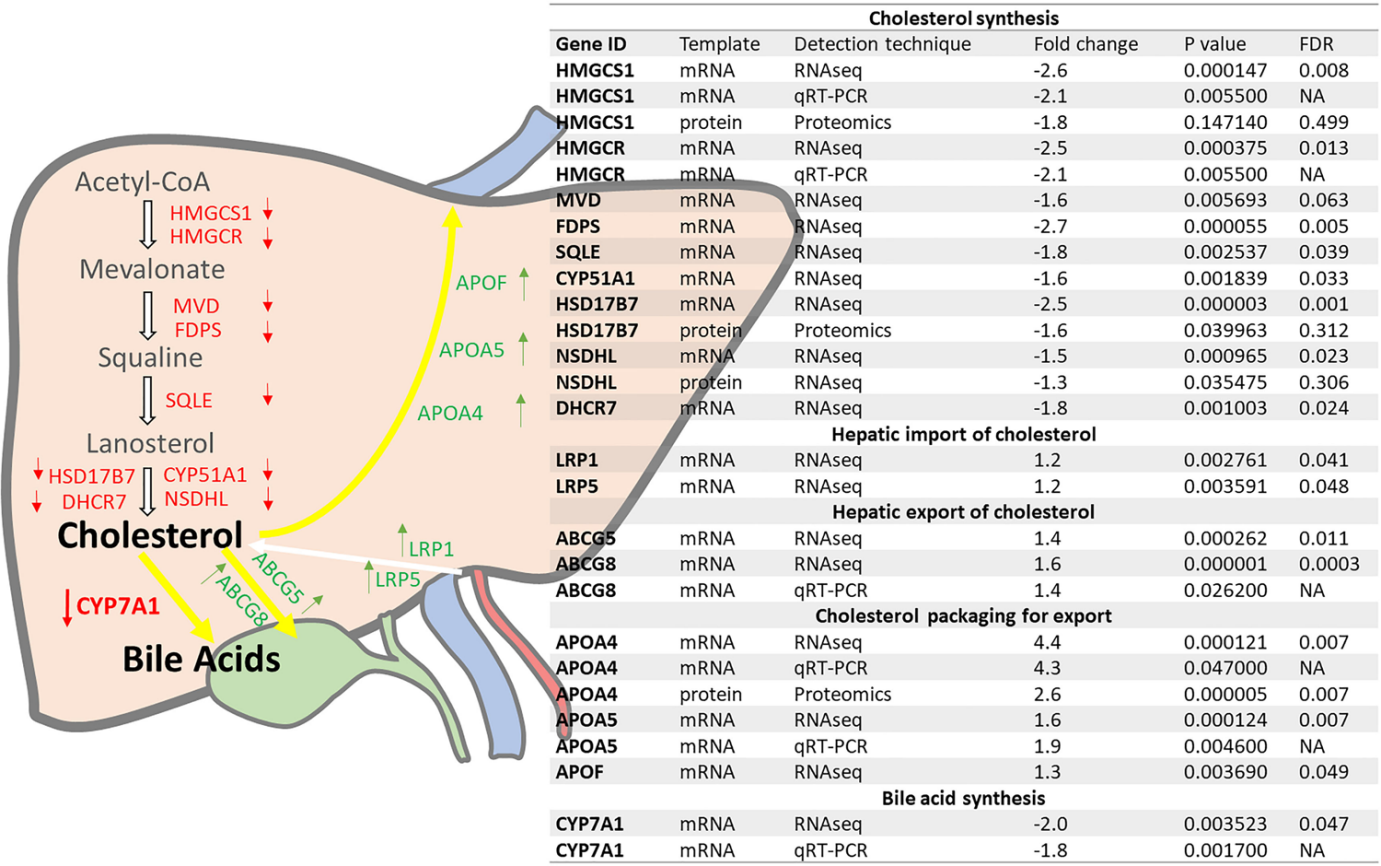
Prenatally androgen exposed male offspring develop hepatic dysregulation of cholesterol and bile acid synthesis. Differentially expressed hepatic genes and proteins between control (C; n = 14) and prenatal androgen excess (PA; n = 14) adolescent offspring relevant to hepatic cholesterol homeostasis. To increase confidence in findings from RNAseq analysis a limited subset of genes was examined by qRT-PCR. In all cases differential expression was similar between RNAseq and qRT-PCR results. Fold change is PA compared to control animals. P values cited are nominal values, false discovery rate adjusted P values (FDR) were calculated as described by Benjamini and Hochberg (1995). Red denotes decreased, green denotes increased in response to prenatal androgen excess.

Analysis of differentially expressed hepatic genes and proteins between control and prenatally androgen treated males revealed a consistent reduction in the cholesterol synthesis pathway (Fig. 2). When genes involved in the hepatic import pathways were analysed the significant differences pointed to an increased potential for cholesterol uptake (Fig. 2). Within the liver there was also increased mRNA encoding genes critical for esterification and assembly of lipoproteins with increased apolipoprotein expression. In addition, gene expression of cholesterol and phospholipid efflux potential into the bile was increased (Table 2). There is thus transcriptional evidence for augmented hepatic pathways involved in cholesterol efflux. However, the hypercholesterolemia in prenatally androgen treated males cannot be explained by increased hepatic cholesterol synthesis or reduced uptake into the liver.

**Table 2:** Differentially expressed hepatic genes and proteins relevant to liver detoxification between control and prenatal-androgen excess pregnancies in male adolescent offspring.

Gene ID	Template	Detection technique	Fold change	P value	FDR
**Liver detoxification potential**					
*GSS*	mRNA	RNAseq	−1.2	0.002081	0.035
*GSS*	mRNA	qRT-PCR	−1.3	0.005800	NA
*GSR*	mRNA	RNAseq	−1.4	0.000131	0.008
*GSTM1*	mRNA	RNAseq	−1.5	0.004742	0.056
*GSTM1*	mRNA	qRT-PCR	−1.3	0.003400	NA
GSTM1	protein	Proteomics	−1.5	0.001069	0.100
GSTM4	protein	Proteomics	−2.8	0.001206	0.100
*GSTO1*	mRNA	RNAseq	−1.3	0.000523	0.016
*GSTO1*	mRNA	qRT-PCR	−1.2	0.015600	NA
GSTO1	protein	Proteomics	−1.2	0.013851	0.213
*UGDH*	mRNA	RNAseq	−1.3	0.001314	0.027
UGDH	protein	Proteomics	−1.2	0.025751	0.274
UGT2B7	protein	Proteomics	−1.3	0.012154	0.200
*UGT3A2*	mRNA	RNAseq	1.2	0.004124	0.052
**ROS detoxification**					
*GPX4*	mRNA	RNAseq	−1.3	0.000187	0.009
GPX4	protein	Proteomics	−1.3	0.022405	0.252
*GPX7*	mRNA	RNAseq	1.7	0.000000006	0.00002
*GPX7*	mRNA	qRT-PCR	1.5	0.000700	NA
*PRDX5*	mRNA	RNAseq	−1.2	0.001224	0.026
PRDX5	protein	Proteomics	−1.2	0.014872	0.221
*TXN*	mRNA	RNAseq	−1.2	0.002934	0.043
TXN	protein	Proteomics	−1.2	0.062923	0.375

Differentially expressed hepatic genes and proteins between control (C; n = 14) and prenatal androgen excess (PA; n = 14) adolescent offspring relevant to liver detoxification. False discovery rate (FDR) was determined by Benjamini and Hochberg (1995) method. Data are fold change in PA animals relative to vehicle treated animals. To increase confidence in findings from RNAseq analysis a limited subset of genes was examined by qRT-PCR. In all cases differential expression was similar between RNAseq and qRT-PCR results. In a small number of selected cases, where a protein has either particular relevance as part of a subset of proteins with related function, or where its gene was found to be differentially regulated, we have reported fold changes of those which were not statistically significant after FDR control (nominal and adjusted *P* values cited in all such cases).

### Dysregulation of the enterohepatic circulation

**Figure 3:**
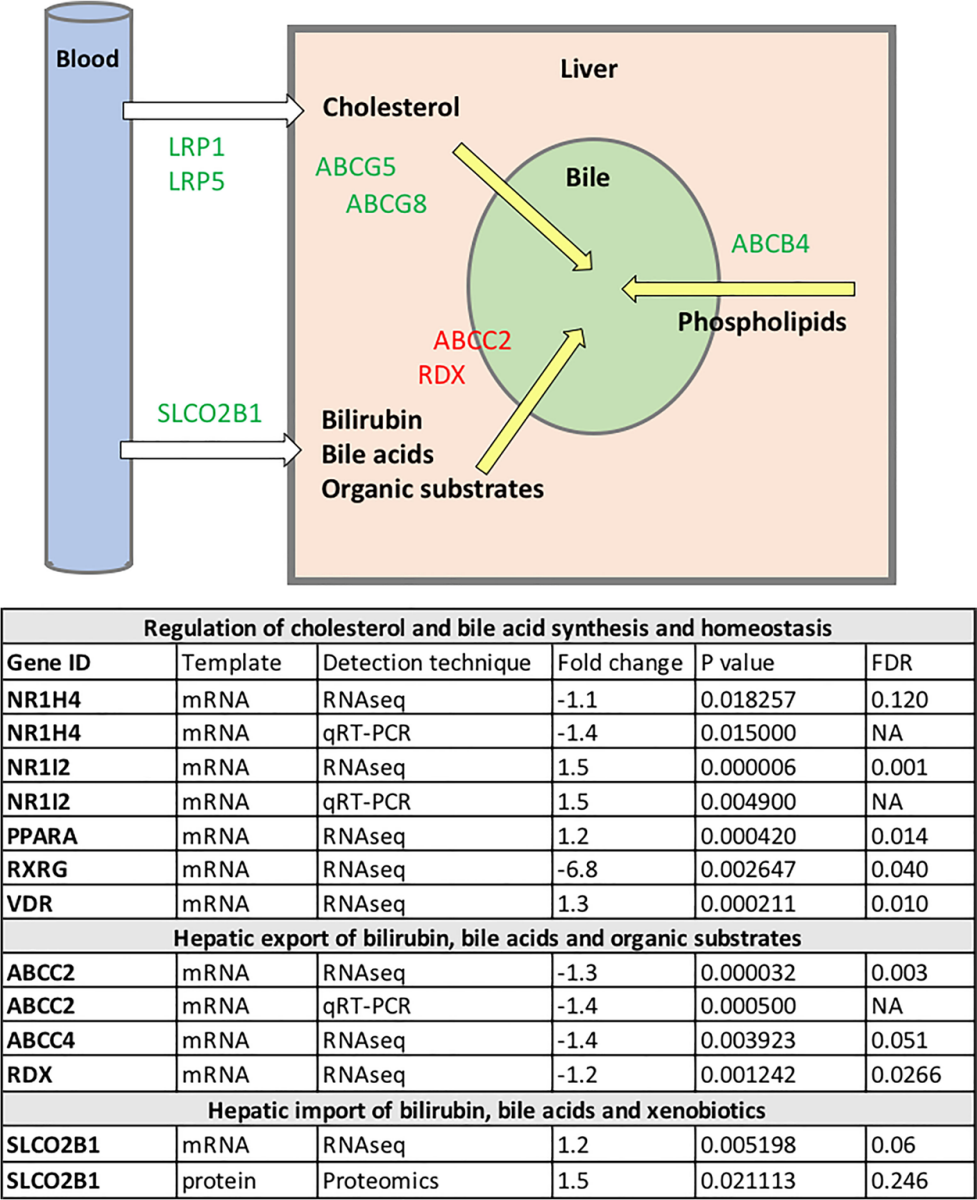
Prenatally androgen exposed develop hepatic dysregulation of cholesterol and bile trafficking.: Enterohepatic circulation. Differentially expressed hepatic genes and proteins between control (C; n = 14) and prenatal androgen excess (PA; n = 14) adolescent offspring relevant to hepatic regulation of cholesterol, bilirubin, bile acids and organic substrates trafficking. False discovery rate (RNAseq and proteomics; FDR) was determined by Benjamini and Hochberg (1995) method. Data are fold change in PA animals relative to vehicle treated animals. To increase confidence in findings from RNAseq analysis a limited subset of genes was examined by qRT-PCR. In all cases differential expression was similar between RNAseq and qRT-PCR results. In a small number of selected cases, where a protein has either particular relevance as part of a subset of proteins with related function, or where its gene was found to be differentially regulated, we have reported fold changes of those which were not statistically significant after FDR control (nominal and adjusted *P* values cited in all such cases).

**Figure 4:**
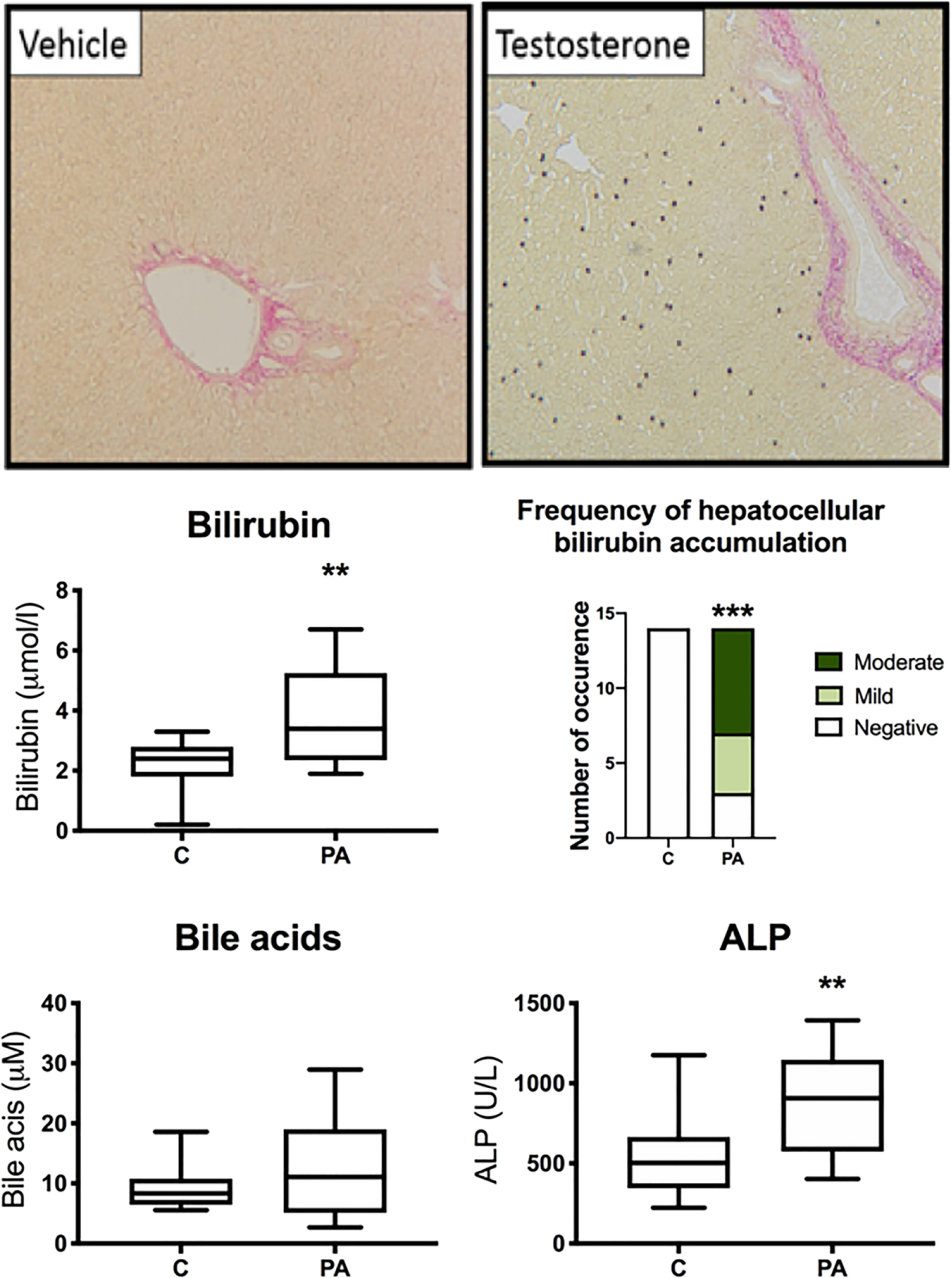
Prenatally androgen exposed male offspring develop increased hepatic bilirubin accumulation, increased circulatory bilirubin concentrations, and increased circulating ALP, without any significant alterations circulating bile acid concentration. Plasma samples collected at 6 months postnatal age from male offspring were assayed for bilirubin, bile acids and ALP; incidence of bilirubin accumulation in liver was determined histologically after oxidisation to biliverdin via Fouchet’s reagent (indicated by black/green punctate staining, upper panels). Increased circulating total bilirubin, hepatic bilirubin accumulation incidence and plasma ALP were noted in PA males. Representative images of sections from each prenatal treatment group are shown (x20 magnification). Differences were analysed by unpaired, two-tailed t-test in plasma, and Chi-square in the case of incidence of hepatic bilirubin accumulation (*P < 0.05; **P < 0.01, ***P < 0.001). Box plot whiskers are lowest and highest observed values, box is the upper and lower quartile, with median represented by line in box.

While there was evidence of increased capacity for cholesterol and phospholipid secretion into the bile, the same was not true for the secretion of bilirubin, bile acids and organic substrates (Fig. 3), predicting increased risk of bilirubin and bile acids accumulation within the liver. Adolescent prenatally androgen treated males had significantly increased hepatic bilirubin content as compared with adolescent controls (*P* < 0.001) (Fig. 4), with foci of accumulation reminiscent of human cholestasis histopathology. Prenatally androgen treated males also exhibited markedly higher circulating concentrations of bilirubin as compared to control pregnancy male offspring during adolescence (3.8 ± 0.44 µmol/l vs 2.27 ± 0.22 µmol/l; *P* < 0.01) (Fig. 4).

Bile acids directly interact with several nuclear receptors within the liver. There were clear effects on genes that mediate bile acid action and homeostasis in prenatally androgen treated males (Fig. 3). While it is difficult to predict the net outcome of these changes it is clear that hepatic bile acid synthesis (via. *CYP7A1*) is likely to be reduced (Fig. 2). Bioinformatic analysis of the hepatic proteome predicted altered bile acid synthesis (Table 1). There was no difference in total circulating bile acids in prenatally androgen treated males (12.79 ± 2.36 µmol/l) as compared to control pregnancy offspring (9.39 ± 1.67µmol/l) (P = 0.16) (Fig. 4). Plasma ALP, known to increase during cholestasis, was increased in PA males (892.9 ± 83.8 U/L) as compared to control males (535.5 ± 67.8 U/L) during adolescence (P < 0.01) (Fig. 4). Bioinformatic analysis of the prenatally androgen treated male hepatic transcriptome predicted intrahepatic cholestasis and analysis of the hepatic proteome also predicted cholestasis (Table 1).

### Reduced hepatic detoxification potential

Hepatic transcriptome analysis identified the potential for liver damage in prenatally androgen treated males. Liver damage, drug-induced liver disease and hepatocyte proliferation pathways were identified by bioinformatics analysis (Table 1). Hepatic proteome bioinformatics also predicted an increase of oxidative stress in livers of adolescent prenatally androgen treated males, accompanied by decreased hydrogen peroxide metabolism and increased synthesis of reactive oxygen species (Table 1). Moreover, there was a prediction of altered concentration of glutathione and increased cell death (Table 1). Bioinformatic analysis of the plasma proteome indicated increased biosynthesis of hydrogen peroxide, metabolism of reactive oxygen species and synthesis of reactive oxygen species (Table 1). There was a general reduction in the expression of hepatic genes and proteins relevant to both liver detoxification potential and liver reactive oxygen species (ROS) detoxification as a consequence of prenatal androgen excess in males (Table 2). Overall the emergent picture is one of reduced hepatic detoxification and potential liver damage.

### Early liver damage

There was no difference between prenatally androgen treated males (14.86 ± 1.67 U/L) and control males (19.46 ± 2.52 U/L) (P = 0.13) in concentrations of plasma alanine aminotransferase (ALT), a marker of hepatocellular damage. This is despite the observation, mentioned above, that ALP was significantly increased in prenatally androgen treated animals. However, the plasma transcriptome predicted fibrosis, liver lesion, hepatic injury and liver cirrhosis (Table 1). We interrogated expression of differentially expressed genes and proteins relevant to liver fibrosis as a potential consequence of liver damage, cell death and proliferation (Table 3). The resulting data suggest that these males have an increased potential for hepatic fibrosis.

**Table 3:** Differentially expressed hepatic genes and proteins relevant to liver fibrosis between control and prenatal-androgen excess pregnancies in male adolescent offspring.

Gene ID	Template	Detection technique	Fold change	P value	FDR
**Fibrosis regulation and signalling**					
*FGF7*	mRNA	RNAseq	1.5	0.000136	0.008
*FGFR2*	mRNA	RNAseq	1.2	0.000974	0.023
*FGFR3*	mRNA	RNAseq	1.3	0.000001	<0.001
*FGFRL1*	mRNA	RNAseq	1.3	0.000034	0.003
*SMAD3*	mRNA	RNAseq	1.3	0.002605	0.040
*SMAD7*	mRNA	RNAseq	1.4	0.000252	0.011
*TGFA*	mRNA	RNAseq	1.2	0.000107	0.007
*TGFBI*	mRNA	RNAseq	1.2	0.003776	0.049
**Fibrosis effectors**					
COL1A1	protein	Proteomics	2.2	0.046590	0.330
*COL4A1*	mRNA	RNAseq	1.3	0.000892	0.022
*COL4A2*	mRNA	RNAseq	1.2	0.007580	0.074
*COL4A4*	mRNA	RNAseq	1.3	0.013936	0.103
*COL4A5*	mRNA	RNAseq	1.8	0.000008	0.001
*COL4A6*	mRNA	RNAseq	10.3	<0.000001	<0.001
*COL6A6*	mRNA	RNAseq	1.3	0.013089	0.099
*COL18A1*	mRNA	RNAseq	1.4	0.000015	0.002
*COL27A1*	mRNA	RNAseq	1.4	0.002068	0.035

Differentially expressed hepatic genes and proteins between control (C; n = 14) and prenatal androgen excess (PA; n = 14) adolescent offspring relevant to liver fibrosis markers. False discovery rate (FDR) was determined by Benjamini and Hochberg (1995) method. Data are fold change in PA animals relative to vehicle treated animals. In a small number of selected cases, where a protein has either particular relevance as part of a subset of proteins with related function, or where its gene was found to be differentially regulated, we have reported fold changes of those which were not statistically significant after FDR control (nominal and adjusted *P* values cited in all such cases).

### Systemic effects

Bioinformatic analysis of the profile of altered plasma proteins in postnatal, adolescent, prenatally androgen treated males was consistent with an increased risk of overall increased morbidity and mortality. Altered pathways included diabetes mellitus, diabetic complications, occlusion of artery and atherosclerosis (Table 1). Indeed, postnatal, adolescent, prenatally androgen treated males had increased levels of fasting plasma insulin (0.55 ± 0.27 ng/ml vs 0.37 ± 0.10 ng/ml; P < 0.05) (Fig. 1A), consistent with a prenatally programmed reduction in health-span in adult males.

### Hepatic expression of selected genes in fetal life

**Figure 5:**
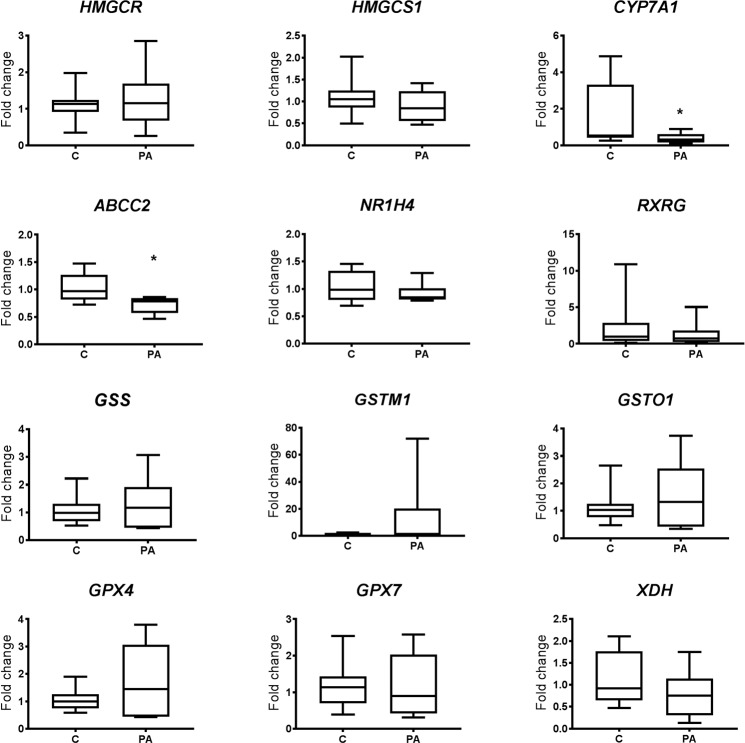
Examination of differentially expressed genes identified in adult life as consequential of prenatal androgen excess by qPCR in fetal livers (day 90 of gestation) during androgen excess treatment. Control (C; n = 10) and prenatal androgen excess (PA; n = 6) male fetal livers were examined by qPCR in terms of genes identified as differentially expressed during adolescence attributable to prenatal androgen status. Statistical testing by unpaired, two tailed Student t test, (*P < 0.05). Of all genes examined, only *CYP7A1* and *ABCC2* mRNA were depressed in fetal life (in addition to postnatal life) by fetal androgen excess. Box plot whiskers are lowest and highest observed values, box is the upper and lower quartile, with median represented by line in box.

Hepatic expression of selected genes (altered in postnatal life by prenatal androgen excess) was investigated in fetal males, on day 90 of pregnancy, exposed to increased levels of androgens and compared to fetal controls. There was no difference in the expression of *HMGCS1, HMGCR, NR1H4, RXRG, GSS, GSTM1, GSTO1, GPX4, GPX7, XDH* however expression of *CYP7A1* and *ABCC2* was significantly decreased in prenatally androgenised males (P < 0.05; Fig. 5).

### Plasma markers of altered liver function during adolescence

We examined the plasma proteome to determine potential readouts of this pattern of dyslipidaemia associated with reduced hepatic cholesterol synthesis and cholestasis. The plasma proteome highlighted intra-hepatic changes associated with cholesterol homeostasis, detoxification potential, hepatic damage and fibrosis (Table 4). Based upon the observation of reduced expression of *CYP7A1* and *ABCC2* in both fetal and postnatal livers from prenatal androgen excess pregnancies (Figs. 5,2 and 3 respectively), we performed correlation analyses between these two permanently differentially expressed genes, circulating cholesterol, and plasma biomarkers of cholesterol homeostasis, ROS detoxification and liver damage/fibrosis (Table 4). Based upon strength of correlation, whilst all plasma protein markers showed strong correlation with circulating cholesterol, the panel of fibrotic proteins, COL1A1, COL1A2, COL2A1 and COL5A1 were the most consistent in terms of robust fold changes in the circulation and association with reduced expression of *CYP7A1* and *ABCC2* genes in the liver (Table 4). This indicated potential utility of circulating monomeric collagen peptides as markers of developing hepatic dysfunction.

**Table 4:** Differentially expressed plasma proteins relevant to cholesterol homeostasis, ROS detoxification potential and liver damage and fibrosis, and their relationships to gene expression altered in both fetal and postnatal life.

Plasma protein	PA fold change	FDR	Plasma Cholesterol		Hepatic *CYP7A1* mRNA		Hepatic *ABCC2* mRNA	
**Cholesterol homeostasis**								
APOA1	1.4	0.344	r 0.53	P 0.0055	r −0.30	NS	r −0.19	NS
APOA2	1.3	0.137	r 0.71	P < 0.0001	r −0.32	NS	r −0.35	NS
APOA4	1.7	0.014	r 0.49	P 0.0044	r −0.23	NS	r −0.41	P = 0.018
APOC3	2.0	0.037	r 0.74	P < 0.0001	r −0.16	NS	r −0.28	NS
APOD	1.8	0.001	r 0.77	P < 0.0001	r −0.32	NS	r −0.44	P = 0.012
APOM	1.5	0.034	r 0.71	P < 0.0001	r −0.39	P = 0.021	r −0.49	P = 0.006
**ROS detoxification potential**								
CAT	−2.8	0.052	r −0.47	P = 0.0171	r 0.55	P = 0.0055	r 0.49	P = 0.014
GPX3	−1.7	0.043	r −0.39	P = 0.022	r 0.39	P = 0.025	r 0.46	P = 0.009
SOD3	−2.0	0.018	r −0.42	P = 0.015	r 0.24	NS	r 0.44	P = 0.011
**Damage/fibrosis**								
COL1A1	2.3	0.044	r 0.55	P = 0.0016	r −0.34	P = 0.046	r −0.62	P = 0.0004
COL1A2	6.1	0.001	r 0.67	P = 0.0001	r −0.45	P = 0.011	r −0.59	P = 0.0009
COL2A1	1.9	0.279	r 0.36	P = 0.043	r −0.55	P = 0.0034	r −0.45	P = 0.015
COL5A1	2.2	0.037	r 0.59	P = 0.0021	r −0.59	P = 0.0025	r −0.67	P = 0.0004

Differentially expressed plasma proteins between control (C; n = 14) and prenatal androgen excess males (PA; n = 14) during adolescence, with relevance as markers of hepatic cholesterol homeostasis, detoxification and fibrotic response were identified. Fold changes are those in PA animals relative to vehicle treated animals. False discovery rate (FDR) was determined by Benjamini and Hochberg (1995) method. In the case of APOA1 we have reported fold change which was not statistically significant after FDR control (nominal and adjusted *P* values cited), since this forms part of a family of apolipoproteins which were significantly altered by prenatal treatment in postnatal adolescent male offspring. Correlation was assessed by calculation of the Pearson r product-moment co-efficient, with P < 0.016 accepted as significant (Bonferroni multiple comparisons adjusted). Collagen proteins, in terms of both fold change in plasma and correlation with CYP7A1, ABCC2 and circulating total cholesterol were the most robust markers of prenatal androgen excess hepatic dysfunction/dyslipidaemia.

## Discussion

Suboptimal intrauterine environments can ‘programme’ adverse postnatal health outcomes. First degree male relatives of hyperandrogenaemic PCOS patients exhibit dyslipidaemia and hyperinsulinaemia^26–28,32^. We investigated whether excess developmental androgen exposure could explain postnatal male dyslipidaemia using an outbred clinically-realistic large-animal model, previously shown to parallel female offspring (fetal androgen excess models and human PCOS daughters) pancreatic function outcomes^23–25,33^. Adolescent prenatally androgenised ovine males had increased circulating concentrations of total cholesterol, HDL-C, LDL-C, TG, AMH and insulin, independent of body weight, adiposity and contemporary testosterone concentrations, thus faithfully reproducing the dyslipidaemic / endocrine profile of first degree male relatives of PCOS patients^26–30^. Neither these observations, nor those discussed below, were recreated by *in utero* estrogenic excess, underscoring the androgen-specific nature of the alterations reported (see Supplementary data set, Fig. 3), and decreasing the likelihood of metabolism of androgen to estrogen as a component mechanism driving observed effects.

Hypercholesterolemia in prenatally androgen treated males contrasted with downregulated cholesterol biosynthesis pathway genes (*HMGCS1, HMGCR, MVD, FDPS, SQLE, CYP51A1, HSD17B7, NSDHL* and *DHCR7)*. Such endogenously suppressed *de novo* cholesterol synthesis, as a negative feedback response to increased circulating cholesterol^34^, provides biologically consistent insight into why statin treatment is less effective^35,36^ in some individuals regarding cholesterol normalisation.

Since we did not find evidence of increased cholesterol synthesis, we focused on cholesterol absorption and excretion. Experimental control and prenatally androgen treated animals were fed a standard diet, with no evidence of increased calorific intake in prenatally androgen treated males, either in terms of feeding behaviour or body weight/adiposity. Adolescent prenatally androgen-treated males had increased expression of hepatic chylomicron remnant receptors *LRP1* and *LRP5*, suggestive of increased hepatic uptake of diet-derived cholesteryl esters^37–39^. However, perhaps as a response to increased cholesterol uptake, these prenatally androgen treated animals also had increased expression of *ABCG5* and *ABCG8* that positively correlated with cholesterol concentrations (r = 0.53; P = 0.002). ABCG5 and ABCG8 are the heterodimer transporter pair that determine clearance of free cholesterol into bile^40,41^. Furthermore, increased biliary secretion of cholesterol underpins predisposition to gallstone formation^42–44^, in turn increasing risk of extrahepatic cholestasis^45^.

Adolescent prenatally androgen treated males also displayed increased concentrations of plasma apolipoproteins, notably APOA4, APOC3, APOD and APOM, in agreement with their dyslipidaemic profile, and in turn strongly correlated with circulating cholesterol. APOA4, a component of HDL and chylomicrons, was also over-represented in liver. APOA4, primarily synthesised in small intestine with only minor amounts derived from liver, reflects dietary lipid absorption^46,47^, and is increased with progression of NAFLD severity^48^. Clearly, elevated plasma APOA4 likely indicates increased small intestinal lipid absorption in prenatally androgen treated animals, supported by a positive relationship between circulating cholesterol and plasma APOA4, increased ABCG5/8, and absence of relationship between plasma APOA4 protein and hepatic APOA4 mRNA (r 0.19, P > 0.05) or protein (r 0.27, P > 0.05). Increased intestinal cholesterol absorption, in combination with decreased cholesterol synthesis, is strongly associated with elevated cardiovascular risk^49,50^. This, in tandem with bioinformatic analysis of plasma protein profiles defining atherosclerosis as a disease risk, reinforces the premise of prenatal excess androgen exposure as a factor in male lifelong health.

Whilst we observed no alterations in circulating bile acid concentrations, our prenatally androgen treated males had decreased hepatic *CYP7A1* expression. *CYP7A1* encodes the rate-limiting enzyme in the classic pathway of cholesterol metabolism to bile acids^51^ (responsible for 90% of bile acid synthesis in humans^52)^. Altered *CYP7A1* expression negatively correlated with total cholesterol (r−0.9, P = 0.004), underscoring the likelihood of decreased metabolism of cholesterol into bile acids, thus offering opportunity for increased secretion / absorption, as witnessed by hepatic *ABCG5/8* profiles and plasma APOA4 increase respectively. Pertinently, *CYP7A1* gene knockout mice have increased total- and LDL-cholesterol^53^, and the human *CYP7A1* homozygous deletion mutation is associated with hypercholesterolemia, HMG-CoA reductase inhibitor resistance and premature coronary disease^54^. Conversely, *CYP7A1* overexpression is characterized by elevated bile acid pool, increased hepatic cholesterol synthesis and lowered serum cholesterol^55,56^. Resveratrol can induce *CYP7A1* expression, increase bile acid pool, reduce hypercholesterolemia^57^ and attenuate liver injury / cholestasis^58,59^, suggesting a potential intervention for prenatally programmed hepatic dysfunction.

Efficient bile acid synthesis is critical for cholesterol homeostasis. Both dyslipidaemia and cholestasis, here predicted by IPA bioinformatics and evidenced by hepatic and circulatory endpoints, may have fetal origins^60^. *CYP7A1* epigenetic modulation during development is an offspring outcome of maternal protein restriction^61,62^, while intra-uterine calorie restriction causes decreased *CYP7A1* expression during fetal and postnatal life, resulting in male-specific hypercholesterolemia^61,62^. We noted that *CYP7A1* was also significantly depressed in fetal livers from prenatally androgen treated males compared to gestational age matched controls. No changes in fetal expression of *de novo* cholesterol synthesis genes were observed, suggesting decreased cholesterol synthesis in adolescents may be consequential of the novel concept of decreased *CYP7A1* expression ‘programmed’ by androgen excess *in utero*.

Adolescent prenatally androgen treated males had increased hepatic expression of *NR1I2* (PXR) and *VDR*, but decreased *NR1H4* (FXR). This in turn may be reflective of altered bile acid signaling^63–66^, due to differential receptor regulation by primary and secondary bile acids^63,65,67^ (partially dependent upon bile acid hydrophobicity^68–70^). Hepatocyte retention of hydrophobic bile acids is causative of liver damage in cholestasis^68^. Furthermore, the role of FXR in bile acid homeostasis, liver regeneration^71^, and cholestatic disease^72,73^ has received significant attention, not least in terms of being a therapeutic target^74–76^. Therefore, this finding of reduced FXR expression is particularly interesting.

*ABCC2* mRNA, encoding MRP2, which mediates canalicular excretion and detoxification of a broad range of compounds, including bilirubin and xenobiotics^77^, was decreased in the livers of our adolescent prenatally androgen treated males. An absence of functional MRP2 from the canalicular membrane is associated with conjugated hyperbilirubinemia and ‘dark pigment’ deposition in hepatocytes in the hereditary *ABCC2* deficiency Dubin-Johnson syndrome (DJS)^78,79^, Eisai hyperbilirubinemic rats^80,81^ and knockout mouse strains^82^. These are in close concordance with our observations of increased plasma bilirubin concentration and hepatic accumulation in our prenatally androgen treated males. Decreased *ABCC2* may be functionally compensated for by upregulation of *ABCC3* (MRP3) and *ABCC4* (MRP4) in DJS patients and animal models of cholestasis^82–85^. However, prenatally androgen treated males showed no compensatory increase in *ABCC3*, while *ABCC4* expression was downregulated. Our data indicated an extremely strong correlation between depressed *ABCC2* and *NR1H4* mRNA (encoding FXR) expression (r 0.91, P < 0.0001). This is in keeping with FXR’s regulatory role over *ABCC2*^86,87^. Adolescent prenatally androgenised males also had decreased expression of *RDX* mRNA (encoding radixin), which is required for MRP2 anchoring in the canalicular membrane^88,89^. *RDX*-deficient mice show increasing serum bilirubin concentrations, culminating in mild liver injury by 8 weeks postnatal age^90^. Although attenuated MRP2 is not of itself a major cause of cholestasis, it is decreased in patients with cholestasis^91–93^, in turn leading to clinical presentations including fibrosis, cirrhosis, liver failure or hepatobiliary malignancy^94^. In addition, our prenatally androgen treated male offspring also displayed increased plasma ALP, in the absence of increased ALT, a common characteristic of cholestasis^95,96^. We conclude, therefore, that an outcome of fetal androgen excess is increased male cholestatic disease risk.

Adolescent prenatally androgen treated males had decreased hepatic expression of GSH synthetase (*GSS*), glutathione reductase (*GSR*), (implying altered oxidative stress response^97^); and glutathione S-transferase genes (*GSTM1*, *GSTM4* and *GSTO1*) (involved in conjugation prior to transport via MRP efflux pumps^98^). Decreased GSH levels are associated with disease states^99^. Such reduced detoxification potential in prenatally androgen treated males was accompanied by increased *PXR* expression. PXR activates phase I drug metabolizing P450 enzymes, phase II drug conjugation enzymes, and phase III drug transporters, including MRP2^100–102^. However, as discussed above, in our study, *ABCC2* and phase II enzyme expression were decreased. PXR (and FXR) form heterodimers with retinoid X receptors RXRA, RXRB and RXRG^103^. Our adolescent prenatally androgen treated males had markedly decreased hepatic *RXRG* expression, which in turn may have impacted detoxification enzyme transcript expression. It is noteworthy that several studies in humans and animal models report decreased glutathione in cholestasis^104–107^. In agreement with our results, expression of proteins UGT2B7 (protein), GSTM1 (mRNA and protein) and GSTM4 (protein) are reduced in obstructive cholestasis^105^, and we extend this panel to include GST01 (mRNA and protein). A similar expression profile of nuclear receptors to that which we observe, with increased PXR, VDR, PPARA and decreased FXR expression^105^, and downregulation of *CYP7A1* expression, was also reported in human obstructive cholestasis^108^. Prenatally androgen treated males also had decreased hepatic glutathione peroxidase 4 (GPX4), peroxiredoxin 5 (PRDX5) and thioredoxin (TXN), antioxidant enzymes that protect cells against oxidative damage^109–111^. In terms of consequential changes linked to altered transcripts/proteins, increased expression of glutathione peroxidase 7 (*GPX7*), an oxidative stress sensor^112^, in our prenatally androgen treated males, in combination with decreased plasma catalase (CAT), glutathione peroxidase 3 (GPX3) and superoxide dismutase 3 (SOD3), yields an antioxidant enzyme profile strongly consistent with chronic liver diseases^113^.

In order to comprehend early life underpinnings of decreased hepatic detoxifying potential, we assessed expression of *ABCC2* and selected detoxifying enzymes in livers from control and prenatally androgen treated fetuses. Interestingly, only *ABCC2* was downregulated in fetal life by androgen excess, indicative that decreased detoxification potential may be secondary to reduced *ABCC2* expression.

Male cholestasis due to anabolic androgenic steroid abuse^114^ is mechanistically associated with increased oxidative stress^115^ and deficiency in hepatic canalicular transporters^116^. Whether this phenomenon is male specific in humans is unknown, but in male rodents, raised testosterone can decrease *MRP2* mRNA and protein, with no effect observed in females^117^. We suggest individualised approaches to treatment, accounting for the stage of cholestatic disease development^118^ may also benefit from understanding of early life contributions to disease development and/or susceptibility.

Early stage markers of different liver diseases, ideally with prognostic value, are acutely required^118^. The utility of new molecular techniques for biomarker discovery is demonstrated by shotgun plasma proteomic assessment of NAFLD^48^. Furthermore, the most robust patho-biochemical signatures identified in a murine model of obstructive cholestasis include increased bilirubin and COL1A1^119,120^, which we also note elevated in circulation here. We observed increased hepatic expression of collagen genes, and increased collagen proteins in plasma of prenatally androgen treated males. We also noted elevated expression of hepatic transforming growth factor beta induced protein (*TGFBI*) (also increased in NAFLD plasma)^48^, *SMAD-3* and -*7, FGF7* (which correlates with hepatic fibrosis severity^121,122^ and suppresses *CYP7A1* during fibrosis development^123)^, *FGFR2* and *FGFR3*. Collectively, this indicated a pro-tissue remodelling/pro-fibrotic hepatic environment developing. In light of collagen-1 as a potential prognostic marker of fibrosis in biliary atresia^124^, we suggest that in combination with circulating cholesterol and bilirubin, COL1A1, COL1A2, COL2A1 and COL5A1 may constitute circulating markers of early stage, pre-symptomatic cholestatic disease development in at risk individuals.

We recognise three major limitations to this work. Only a single postnatal time point was studied; however, we see no suggestions here that the condition we describe was likely to spontaneously resolve in later life. We acknowledge species difference between humans and sheep, and that a single dose of testosterone was used, nonetheless, our data closely parallels human clinical outcomes (dyslipidaemia and hormonal alterations) of hyperandrogenaemic pregnancy conditions, and mechanistic investigations performed in laboratory rodent models of hepatic disease

In conclusion, the legacy of male fetal androgenic excess is the development of a cholestasis-like condition in adolescence, accompanied by dyslipidaemia and increased fibrotic potential. We report fetal androgen excess as a risk factor for poor male hepatic/metabolic health.

## Materials and Methods

### Statistical analysis

All experiments were carried out on n = 14 male offspring per group, except in the case of a limited estrogenic overexposure comparison (see Supplementary Materials), and in the case of fetal tissue analyses (actual numbers described in methods and results sections). No animals or data were excluded from any of the analyses described. RNAseq and proteomic data was examined by pairwise comparisons, using Benjamini and Hochberg (1995) false discovery rate control. In cases of single gene analyses, all data sets were normality tested prior to further analysis (Shapiro-Wilk test), and logarithmically transformed if necessary. For comparing means of two treatment groups with equal variances, unpaired, two-tailed Student’s t test was used accepting *P* < 0.05 as significant. Bonferroni multiple test correction was utilised in the case of data subjected to t-test. A Chi-square test was used for testing categorical variables. Correlation was assessed by calculation of Pearson product-moment co-efficient. Statistical analysis software package R (version 3.4.0) was utilised for all RNAseq and proteomic analyses, and the remainder of statistical analysis was performed using GraphPad Prism 8.0 software (GraphPad Prism Software, San Diego, CA, USA). All *P* values are cited in full.

### Study approval

All studies were approved by the UK Home Office and conducted under approved Project Licence PPL 60/4401, reviewed by The University of Edinburgh Animal Research Ethics Committee.

### Animals and tissue collection

All animal based components of the study were conducted under natural lighting conditions with no environmental manipulations. Animal husbandry, experimental protocols and tissue collection were performed as are exactly as previously described^23,24^. Briefly, mature Scottish Greyface ewes were fed in order to achieve a comparable body condition score (2.75–3) prior to estrous cycle synchronisation. After a synchronised mating (Texel ram), animals were allocated at random to one of two experimental groups – vehicle control or testosterone propionate (TP) exposed. Treatment occurred on day 62 and day 82 of gestation (post-sexual differentiation, designed to maintain elevated testosterone concentrations over experimental period^23,24)^. Anaesthesia was induced on day 62 of gestation by initial sedation via administration of 10 mg Xylazine i.m (‘Rompun’, Baylor plc Animal Health Division, UK), followed by 2 mg/kg ketamine (i.v, Keteset, Fort Dodge Animal Health, UK). All downstream procedures were conducted under surgical aseptic conditions. TP was dissolved in vegetable oil (100 mg/ml) and a 200 μl volume injected (20 G Quinke spinal needle, BD Biosciences) via ultrasound guidance into the fetal flank (intraperitoneal injection). Control fetuses received 200 μl vegetable oil vehicle alone. In order to account for potential aromatisation of testosterone to estrogen, and thus incorrect interpretation of effects being attributable to androgenic excess, an additional treatment group of estrogenic overexposure (n = 8) was created by exposing fetuses, in an identical manner to androgen excess exposure, by injection of 200 µl of 20 mg/ml DES (diethylstilbesterol), this lower dose as compared to testosterone being selected on the basis of binding affinity of DES as compared to the naturally occurring estradiol receptor ligand estradiol-17β^125^. These procedures were repeated on d82 of gestation. Immediately after surgical procedure completion all pregnant ewes were given prophylactic antibiotics (Streptacare, Animalcare Ltd, UK, 1 ml/25 kg) and were then monitored during recovery; no adverse effects of these procedures were observed.

Fetal tissue collection was as previously described, performed on day 90 of gestation (day 147 is term)^23,24^.

Offspring were lambed and reared conventionally (weaned at 3 months postnatal age). At birth, and during postnatal life, we saw no phenotypic evidence of any deleterious effects of the treatment regimens used. In female children of PCOS sufferers, early puberty (Tanner 1 developmental stage) is when measurable metabolic perturbations (hyperinsulinaemia) begin to manifest, hence the 6 months postnatal age studied here, as an equivalent of the human observations^126^. Prior to sacrifice at 6 months of age, all animals received a bolus injection of glucose (10 g glucose in 20 ml saline), and 15 minutes post injection sacrifice was achieved via barbiturate overdose. Animals were sacrificed in a random order with respect to treatments. Tissues were recovered immediately, and snap frozen prior to storage at −80 °C until downstream analysis.

In both fetal and postnatal tissue collection, liver sampling occurred from the same lobe (right posterior), in approximately the same area, and was immediately snap frozen, then stored at −80 °C until further processing in the case of nucleic acid and protein extraction, or fixed and processed as previously described^23,24^. These procedures were repeated over two breeding seasons, maintaining all conditions identically, to ensure sufficient numbers of male offspring for analysis.

### RNA and protein extraction

RNA and protein were extracted and purified using Qiagen AllPrep kits, following manufacturer’s instructions. All samples were randomised within a larger study set to control for potential batch effects. RNA concentrations were determined using a NanoDrop 1000 spectrophotometer (Thermo Fisher Scientific, UK), with Agilent Bioanalyser analysis utilised for RNA quality control – all samples processed for downstream analysis recorded a RIN value of >7.5. RNA and protein extracts were stored at −80 °C until further analysis.

### RNA sequencing transcriptomic analyses

1 μl of ERCC (External RNA Controls Consortium) spike in controls were added to 500 ng of RNA sample to permit library quality assessment and estimation of lowest limit of detection^127^. One of the two ERCC mixes were selected at random for each sample. Libraries were prepared with the Illumina TruSeq Stranded mRNA kit, using fetal-vehicle treated (control) liver samples (n = 14) and fetal-testosterone propionate treated liver samples (n = 14), sampled at 6 months postnatal age. Sequencing was performed on the NextSeq. 500 High Output v2 kit (75 cycles) on the Illumina NextSeq. 500 platform producing 75 bp single end reads. Raw sequencing data is available from the ArrayExpress database (http://www.ebi.ac.uk/arrayexpress) under accession number E-MTAB-8032. To assess quality of sequencing data, reads were analysed with FastQC (version 0.11.3)^128^. To remove any lower quality and adapter sequences, TrimGalore! (version 0.4.0)^129^ was used to filter the reads (phred quality score threshold of 30). To remove the ERCC reads, all reads were aligned to the ERCC reference genome using HISAT2 (version 2.1.0)^130^. These alignments were processed using SAMtools (version 1.2)^131^, reads were counted using featureCounts (part of the sub read version 5.0.1 package)^132^ and analysed using the R package erccdashboard (version 1.6.0)^133^.

The most recent release of the sheep *Ovis aries* reference genome was downloaded from NCBI (https://www.ncbi.nlm.nih.gov/genome?term = ovis%20aries)^134^ along with the equivalent annotation file, and reads were aligned to this reference using HISAT2 (version 2.1.0)^130^ with the parameter for stranded library preparation used. SAMtools (version 1.2)^131^ was used to process the alignments and reads were counted at gene locations using featureCounts (part of the sub read version 5.0.1 package)^132^ utilising the parameter to split multi-mapped reads as a fraction across all genes that they align to. Pairwise gene comparisons were carried out with edgeR (version 3.16.5)^135^ with all genes with CPM (count per million) value of more than one in six kept for analysis, and all other genes removed as low count genes, leaving 15134 genes for analysis. P values were adjusted using the Benjamini-Hochberg procedure, with a false discovery rate (FDR) set at q < 0.05^136^.

### Liver and plasma protein quantification

Hepatic and plasma proteins were identified and quantified using a Q Exactive Plus hybrid quadrupole Orbitrap mass spectrometer fitted with an EASY-Spray nano-ESI source (Thermo Scientific) as previously described^137^. Briefly, 10 micrograms of proteins were diluted in 100 μl of 50 mM NH_4_HCO_3_ (BioUltra grade, Sigma Aldrich), and were reduced, alkylated and digested with trypsin overnight according to the PRIME-XS protocol (http://www.primexs.eu/protocols/Public-Documents/04%2D%2D-Protocols/PRIME-XS-Protocol-NPC-In-Solution Digestion.pdf/). The equivalent of 2 μg of peptides (assuming no losses) were analyzed by liquid chromatography tandem mass spectrometry (LC-MS/MS). All peptide matching searches were performed against FASTA file of the ovine, bovine, swine, equine, and caprine proteomes (canonical and isoform sequences retrieved from Uniprot on the 22^nd^ of February 2018). Match between runs was used to identify peptide signals lacking MS/MS information. Protein intensities across samples were normalized using the maxLFQ algorithm^138^. Normalised protein intensities for all proteins were then extracted from the results file for the downstream statistical analyses. Pairwise protein comparisons were carried out using *limma*^139^ for those proteins that yielded normalised intensities in at least 75% of the compared samples. P values were adjusted using the Benjamini-Hochberg procedure^136^. Mass spectrometry proteomics data have been deposited to the ProteomeXchange Consortium via the PRIDE^140^ partner repository with the dataset identifier PXD014050.

### Quantitative PCR

Quantitative PCR was performed exactly as previously described^23^, utilising Genorm analysis (PrimerDesign Ltd, UK) in order to identify a panel of two stable housekeeping genes – thus the geometric mean of *ATPsynth* and *RPL19* was used as the normalisation reference. Negative controls consisted of an RT-ve and a template negative reaction. Primers were designed in house using the Primer3Plus bioinformatics software tool, were synthesised by Eurofins MWG Operon, Germany, and were validated prior to use (Supplementary Table 1), or were outsourced and designed by PrimerDesign Ltd, (UK).

### Bioinformatic analysis

IPA (Ingenuity Pathway Analysis, Qiagen) was used to perform initial screens of affected pathways, and to identify potential disease states associated with the differentially expressed genes (DEG’s) and differentially expressed proteins (DEP’s). Pre-filtering of data uploaded to IPA was based upon FDR adjusted significance, using *q* < 0.05 as cut-off.

IPA *P* values of <0.05 (Right-tailed Fishers exact test) delivered information regarding association of significant DEG with biological processes, and directional change with regards to such biological pathways in health and disease generated where possible. Given smaller numbers of proteins identified, in order to generate meaningful bioinformatics analyses, we relaxed the stringency of input by using nominal P values of <0.05.

### Plasma analyte determination

Concentrations of fasting plasma total cholesterol, high density lipoprotein (HDL), Triglyceride (TG) total bilirubin, alanine aminotransferase (ALT) and alkaline phosphatase (ALP) were obtained using commercial assay kits (Alpha Laboratories Ltd., Eastleigh, UK) as per manufacturer’s instruction, using a Cobas Mira automated analyser (Roche Diagnostics Ltd, UK). Low density lipoprotein (LDL) was calculated by the Friedewald equation^141^. For all assays within-run and intra-batch precisions were <4% CV and <5% CV, respectively. Fasting plasma bile acid concentration was measured using Total Bile Acid Assay Kit (STA-631; Cell Biolabs, INC., San Diego, US), as per the manufacturer’s instructions with all samples assayed in duplicate. The assay sensitivity was 0.39 µM and intra-assay CV was <4%. Plasma insulin was measured using the ALPCO Ovine Insulin Elisa kit (80-INSOV-E01; American Laboratory Products Company, Salem, US) as per the manufacturer’s instructions, with all samples measured in duplicate. The assay sensitivity was 0.15 ng/ml and intra-assay CV was <4.5%. Testosterone was measured by in-house ELISA. Briefly, 96-well plates (Greiner Bio-One GmbH, Germany) were coated with 100 µl of donkey anti rabbit IgG (Jackson ImmunoResearch Inc, USA) (1:500 in ELISA coating buffer, 100 mM Sodium Bicarbonate, pH 9.6) and incubated (4 °C, 12 hours). After washing twice (0.05 M Tris/HCl + 0.05% Tween 20, pH 7.4), 250 μl of Blocking buffer was added and incubated for 1 h (room temperature with shaking), then washed twice. Standards (0–24.3 ng/ml), samples and controls (20 µl per well) were added to each well, followed by 80 µl of Testosterone-HRP conjugate (Astra Biotech GmbH, Germany) at 1:20,000 in assay buffer (PBS pH 7.4, 0.1%BSA with 250 ng/ml cortisol (to displace any testosterone bound to binding globulins)), followed by 50 μl of Rabbit anti Testosterone −19 antibody (AMS Biotechnology, USA) (1:200,000). Plates were incubated (room temperature, 2 hours) then washed 5 times. 120 µl of substrate solution (3,3,5,5-Tetramethylbenzidine, Millipore Corporation, USA) was added and incubated (room temperature, in darkness, 20 min). Reaction was stopped by adding 80 µl of 2 N H_2_SO_4_ solution, and. plates read at 450 nm. The inter-assay CV for low and high testosterone QC pools were 11.4 and 9.1% respectively; intra-assay CV’s were 8.9 and 5.6%, with detection limit of 0.1 ng/ml. Dihydrotestosterone cross-reacted 20.4%, all other tested steroids showed <0.2% cross-reactivity.

### Hepatic bilirubin content

Wax embedded tissue sections (5 μm; 2 per sample), were mounted on to charged slides (Superfrost Plus, Thermoscientific, Epsom, UK), then rehydrated through a graded alcohol series. Halls Bilirubin stain was then performed, using Fouchets and Van Giesons reagents (Sigma Aldrich, Poole, Dorset) following an online protocol (https://webpath.med.utah.edu/HISTHTML/MANUALS/BILE.PDF). Assessment by light microscopy was performed by two independent operators, both blinded to treatment. Scoring was semiquantitive, categorical based, where 0 = no bilirubin deposits, 1 = mild and 2 = intense bilirubin deposits).

## Supplementary information

Supplementary information.
