# Supplementary material for: Fetal androgen exposure is a determinant of adult male metabolic health
Source: Sci Rep. 2019 Dec 27;9:20195. doi: 10.1038/s41598-019-56790-4 (PMC6934666; doi:10.1038/s41598-019-56790-4)
Supplement: Supplementary file 1 — Supplementary information. [file 41598_2019_56790_MOESM1_ESM.docx]

**Supplementary data**

**Table 1.** Forward and reverse primer sequences and amplicon size for genes analysed in the fetal and postnatal hepatic samples using qRTPCR.

| **Gene** | **Forward Sequence** | **Reverse Sequence** | **Product Size (bp)** |
| --- | --- | --- | --- |
| *ABCC2* | CAGCGATTGACAACAACCAG | CCCACAGTGTCCCCATTTAG | 154 |
| *APOA4* | ACGGCGAGACCTTCAACAAA | CCCAAAGGGACGGGTATCTC | 208 |
| *APOA5* | CCGAGAGACTGAGCAAGTCC | GATGTCTTCCCACAGGTCGT | 121 |
| *CYP7A1* | ACCTGCAGACTGATGGGAAA | ATCACTCGGGTCAATGCTTC | 153 |
| *GPX4* | GATCTGTGTAAATGGGGACGA | GCTCTTCCATGGGACCATAC | 161 |
| *GPX7* | TTCAAGGCCGTCAACATCCG | ACTCGCTAGCCACATTCACC | 88 |
| *GSS* | CCACTTTTGCTGGCCTCTAC | CTGAGGCTTTAGCACGAAGC | 101 |
| *GSTM1* | TCCTGGGTTACTGGGACATC | CGTCCCCCATTGTGTACTTC | 102 |
| *GSTO1* | CCTGATCCGTGTCTACAGCA | GACCCTGACTGGTTTCCAGA | 176 |
| *HMGCR* | TCGAGATCATGTGCTGCTTC | CTAAGACTCGGGCAAAATGG | 159 |
| *HMGCS1* | AGGATCGGCGTGTTTTCTTA | TCCTCTCGGAGCTTCATGTT | 191 |
| *NR1H4* | CCTGTGAGGGATGCAAAGGT | AGCATTCAGCCAACATTCCC | 162 |
| *RXRG* | ATGAAGATATGCCCGTGGAG | GGCCCATTCAACGAGAGTAA | 161 |
| *XDH* | AGGAGTTTATGCCCCTGGAT | GAGCTTTGAGGTCCAGCAAC | 156 |

**Table 2. RNAseq read statistics of all adolescent offspring samples analysed in study.** PA = prenatal androgen excess samples). E = diethylstilbesterol exposed (prenatal estrogen excess samples) (see materials and methods).

| **Sample** | **Treatment** | **# of Raw Reads** | **# of Filtered Reads** | **% Filtered reads aligned to ERCC** | **% Filtered reads aligned to *O. aries*** | **% of reads counted at genes** |
| --- | --- | --- | --- | --- | --- | --- |
| 1 | Control | 29139856 | 28947432 | 0.41% | 94.0% | 79.4% |
| 2 | Control | 26998873 | 26834790 | 0.38% | 94.5% | 79.2% |
| 3 | Control | 22984307 | 22831776 | 0.32% | 94.7% | 82.1% |
| 4 | Control | 29633699 | 29480827 | 1.09% | 93.8% | 79.0% |
| 5 | Control | 28273358 | 28107325 | 0.92% | 93.2% | 79.5% |
| 6 | Control | 28289429 | 28116354 | 0.30% | 91.4% | 74.2% |
| 7 | Control | 26789367 | 26633721 | 0.40% | 94.6% | 81.5% |
| 8 | Control | 31934466 | 31747323 | 0.81% | 93.6% | 79.6% |
| 9 | Control | 33567791 | 33366876 | 0.95% | 93.5% | 79.6% |
| 10 | Control | 27375312 | 27217565 | 0.36% | 94.4% | 81.0% |
| 11 | Control | 30062008 | 29900312 | 0.48% | 94.7% | 80.6% |
| 12 | Control | 28197830 | 28034317 | 1.10% | 93.3% | 78.4% |
| 13 | Control | 27308728 | 27135675 | 0.92% | 94.2% | 79.8% |
| 14 | Control | 28901373 | 28731822 | 1.40% | 92.2% | 78.1% |
| 38 | PA | 32535305 | 32345627 | 0.32% | 94.1% | 80.4% |
| 39 | PA | 32156322 | 31879445 | 0.36% | 93.9% | 80.4% |
| 40 | PA | 32869396 | 32659613 | 0.30% | 94.1% | 80.8% |
| 41 | PA | 32523321 | 32352832 | 0.89% | 92.9% | 78.7% |
| 42 | PA | 31249221 | 31057930 | 0.96% | 93.5% | 78.8% |
| 43 | PA | 37264418 | 37020504 | 0.37% | 94.6% | 81.6% |
| 44 | PA | 33001822 | 32825752 | 0.41% | 94.0% | 80.1% |
| 45 | PA | 30711458 | 30534545 | 0.88% | 93.8% | 81.1% |
| 46 | PA | 32945874 | 32686640 | 0.92% | 93.4% | 79.2% |
| 47 | PA | 36063108 | 35823258 | 0.37% | 94.5% | 81.1% |
| 48 | PA | 31965279 | 31739645 | 0.40% | 93.8% | 80.5% |
| 49 | PA | 32190494 | 32017613 | 1.04% | 94.4% | 81.2% |
| 50 | PA | 33643343 | 33455089 | 0.90% | 94.0% | 80.8% |
| 51 | P | 35323034 | 35145413 | 1.04% | 93.9% | 79.2% |
| 30 | E | 32192945 | 32015604 | 0.49% | 93.1% | 78.0% |
| 31 | E | 29046687 | 28882124 | 0.33% | 94.3% | 80.6% |
| 32 | E | 33097763 | 32926110 | 0.90% | 93.7% | 80.6% |
| 33 | E | 32753714 | 32537818 | 0.44% | 93.6% | 79.5% |
| 34 | E | 32768552 | 32594471 | 1.15% | 93.5% | 79.4% |
| 35 | E | 31987000 | 31807265 | 0.83% | 93.9% | 80.2% |
| 36 | E | 33435003 | 33258640 | 0.39% | 94.9% | 80.2% |
| 37 | E | 33647971 | 33441562 | 1.45% | 93.4% | 79.9% |

**Table 3. Prenatal estrogenic excess did not affect genes and proteins reported to be altered by prenatal androgen excess** Hepatic genes, proteins, and circulating proteins, identified as differentially expressed between control and prenatal androgen excess were screened in samples exposed to prenatal estrogen excess (see materials and methods) (n=8) adolescent offspring. There were no significant effects of prenatal estrogen excess in any of the genes and proteins found to be altered in adolescence by prenatal androgen excess.

| Cholesterol synthesis | | | | | |
| --- | --- | --- | --- | --- | --- |
| Gene ID | **Template** | **Detection technique** | **Fold change** | **P value** | **FDR** |
| *HMGCS1* | mRNA | RNAseq | -1.2 | 0.612846 | 0.998 |
| HMGCS1 | protein | Proteomics | 1.2 | 0.600977 | 0.927 |
| *HMGCR* | mRNA | RNAseq | -1.0 | 0.905562 | 0.998 |
| *MVD* | mRNA | RNAseq | 1.1 | 0.775538 | 0.998 |
| *FDPS* | mRNA | RNAseq | -1.1 | 0.722661 | 0.998 |
| *SQLE* | mRNA | RNAseq | 1.1 | 0.620113 | 0.998 |
| *CYP51A1* | mRNA | RNAseq | 1.0 | 0.905825 | 0.998 |
| *HSD17B7* | mRNA | RNAseq | -1.2 | 0.524655 | 0.998 |
| HSD17B7 | protein | Proteomics | -1.4 | 0.054855 | 0.872 |
| *NSDHL* | mRNA | RNAseq | 1.0 | 0.961413 | 0.998 |
| NSDHL | protein | RNAseq | 1.0 | 0.777004 | 0.958 |
| *DHCR7* | mRNA | RNAseq | -1.1 | 0.741918 | 0.998 |
| Hepatic import of cholesterol | | | | | |
| *LRP1* | mRNA | RNAseq | -1.0 | 0.617785 | 0.998 |
| *LRP5* | mRNA | RNAseq | 1.0 | 0.686024 | 0.998 |
| Hepatic export of cholesterol | | | | | |
| *ABCG5* | mRNA | RNAseq | -1.0 | 0.723485 | 0.998 |
| *ABCG8* | mRNA | RNAseq | 1.0 | 0.828225 | 0.9983 |
| Hepatic export of phospholipids | | | | | |
| *ABCB4* | mRNA | RNAseq | 1.3 | 0.043288 | 0.988 |
| Bile acid synthesis | | | | | |
| *CYP7A1* | mRNA | RNAseq | -1.0 | 0.955194 | 0.998 |
| Cholesterol packaging for export | | | | | |
| *APOA4* | mRNA | RNAseq | -2.7 | 0.035185 | 0.981 |
| APOA4 | protein | Proteomics | 1.1 | 0.606619 | 0.930 |
| *APOA5* | mRNA | RNAseq | -1.2 | 0.325352 | 0.998 |
| *APOF* | mRNA | RNAseq | 1.0 | 0.801790 | 0.998 |
| Bile acid synthesis | | | | | |
| *CYP7A1* | mRNA | RNAseq | -1.0 | 0.955194 | 0.998 |
| Regulation of cholesterol and bile acid synthesis and homeostasis | | | | | |
| *NR1H4* | mRNA | RNAseq | -1.0 | 0.751151 | 0.998 |
| *NR1I2* | mRNA | RNAseq | 1.0 | 0.695236 | 0.998 |
| *PPARA* | mRNA | RNAseq | 1.0 | 0.679656 | 0.998 |
| *RXRG* | mRNA | RNAseq | 1.7 | 0.416625 | 0.998 |
| *VDR* | mRNA | RNAseq | 1.2 | 0.099671 | 0.998 |
| Hepatic export of bilirubin, bile acids and organic substrates | | | | | |
| *ABCC2* | mRNA | RNAseq | -1.0 | 0.734572 | 0.998 |
| *ABCC4* | mRNA | RNAseq | -1.4 | 0.012533 | 0.967 |
| *RDX* | mRNA | RNAseq | -1.0 | 0.615711 | 0.998 |
| Hepatic import of bilirubin, bile acids and organic substrates | | | | | |
| SLCO2B1 | mRNA | RNAseq | 1.1 | 0.235747 | 0.998 |
| *SLCO2B1* | protein | Proteomics | 1.4 | 0.022728 | 0.859 |
| Liver detoxification potential | | | | | |
| *GSS* | mRNA | RNAseq | -1.1 | 0.216881 | 0.998 |
| *GSR* | mRNA | RNAseq | -1.3 | 0.016578 | 0.968 |
| *GSTM1* | mRNA | RNAseq | -1.0 | 0.790211 | 0.998 |
| GSTM1 | protein | Proteomics | -1.1 | 0.879458 | 0.977 |
| GSTM4 | protein | Proteomics | -1.3 | 0.323604 | 0.900 |
| *GSTO1* | mRNA | RNAseq | -1.0 | 0.749068 | 0.998 |
| GSTO1 | protein | Proteomics | -1.0 | 0.772727 | 0.958 |
| *UGDH* | mRNA | RNAseq | -1.1 | 0.349041 | 0.998 |
| UGDH | protein | Proteomics | -1.2 | 0.097120 | 0.872 |
| UGT2B7 | protein | Proteomics | -1.1 | 0.293500 | 0.893 |
| *UGT3A2* | mRNA | RNAseq | 1.1 | 0.365960 | 0.998 |
| ROS detoxification | | | | | |
| *GPX4* | mRNA | RNAseq | -1.1 | 0.145336 | 0.998 |
| GPX4 | protein | Proteomics | data not available | | |
| *GPX7* | mRNA | RNAseq | 1.1 | 0.610186 | 0.99830 |
| *PRDX5* | mRNA | RNAseq | -1.1 | 0.447523 | 0.998 |
| PRDX5 | protein | Proteomics | -1.1 | 0.089086 | 0.872 |
| QDPR | protein | Proteomics | -1.1 | 0.284650 | 0.893 |
| *TXN* | mRNA | RNAseq | -1.1 | 0.370359 | 0.998 |
| TXN | protein | Proteomics | -1.1 | 0.111980 | 0.872 |
| Fibrosis regulation and signalling | | | | | |
| *FGF7* | mRNA | RNAseq | 1.1 | 0.292744 | 0.998 |
| *FGFR2* | mRNA | RNAseq | 1.2 | 0.008684 | 0.927 |
| *FGFR3* | mRNA | RNAseq | 1.1 | 0.100885 | 0.998 |
| *FGFRL1* | mRNA | RNAseq | -1.0 | 0.935647 | 0.998 |
| *SMAD3* | mRNA | RNAseq | -1.0 | 0.701509 | 0.998 |
| *SMAD7* | mRNA | RNAseq | 1.3 | 0.029607 | 0.968 |
| *TGFA* | mRNA | RNAseq | 1.1 | 0.134859 | 0.998 |
| *TGFBI* | mRNA | RNAseq | 1.1 | 0.265524 | 0.998 |
| Fibrosis effectors | | | | | |
| COL1A1 | protein | Proteomics | 1.1 | 0.629917 | 0.998 |
| *COL4A1* | mRNA | RNAseq | -1.0 | 0.958840 | 0.998 |
| *COL4A2* | mRNA | RNAseq | 1.0 | 0.905461 | 0.998 |
| *COL4A4* | mRNA | RNAseq | 1.2 | 0.099488 | 0.998 |
| *COL4A5* | mRNA | RNAseq | 1.1 | 0.451200 | 0.998 |
| *COL4A6* | mRNA | RNAseq | 1.5 | 0.225751 | 0.998 |
| *COL6A6* | mRNA | RNAseq | 1.1 | 0.623779 | 0.998 |
| *COL18A1* | mRNA | RNAseq | 1.1 | 0.351233 | 0.998 |
| *COL27A1* | mRNA | RNAseq | -1.0 | 0.972519 | 0.998 |
| Cholestreol homeostasis | | | | | |
| APOA1 | plasma | Proteomics | -1.0 | 0.983820 | 0.999 |
| APOA2 | plasma | Proteomics | 1.0 | 0.803849 | 0.991 |
| APOA4 | plasma | Proteomics | 1.0 | 0.786092 | 0.991 |
| APOC3 | plasma | Proteomics | -1.5 | 0.084049 | 0.738 |
| APOD | plasma | Proteomics | 1.2 | 0.261051 | 0.897 |
| APOM | plasma | Proteomics | -1.0 | 0.960485 | 0.999 |
| ROS detoxification potential | | | | | |
| CAT | plasma | Proteomics | -1.5 | 0.442702 | 0.934 |
| GPX3 | plasma | Proteomics | -1.5 | 0.058539 | 0.679 |
| SOD3 | plasma | Proteomics | 1.0 | 0.988229 | 0.999 |
| Hepatic damage and fibrosis markers | | | | | |
| COL1A1 | plasma | Proteomics | 1.0 | 0.97030 | 0.999 |
| COL1A2 | plasma | Proteomics | 1.1 | 0.87725 | 0.999 |
| COL2A1 | plasma | Proteomics | -1.3 | 0.34340 | 0.900 |
| COL5A1 | plasma | Proteomics | data not available | | |
